# Chloroplast genome sequencing and phylogenetic analysis of *Brassica tournefortii* Gouan (Brassicaceae)

**DOI:** 10.1080/23802359.2025.2529842

**Published:** 2025-07-11

**Authors:** Eun Su Kang, Dong Chan Son, Sang-Chul Kim

**Affiliations:** Forest Biodiversity Division, Korea National Arboretum, Pocheon-si, Gyeonggi-do, Republic of Korea

**Keywords:** Plastome, coding sequences, *B*rassica, phylogenetic relationship

## Abstract

This study reported the chloroplast genome of *Brassica tournefortii* Gouan (Brassicaceae). The chloroplast genome was 153,167 bp, including a large single copy region of 83,247 bp, a small single copy region of 17,816 bp, and two inverted repeat regions of 26,052 bp. It contains 129 genes, comprising 84 coding sequences (CDSs), 37 tRNAs, and 8 rRNAs. Phylogenetic analysis of 78 CDSs showed *B. tournefortii* closely related to *B. carinata* A.Braun and *B. nigra* (L.) W.D.J.Koch. These findings provide essential chloroplast genome data for *B. tournefortii*, supporting research on species identification, evolutionary studies, and understanding species invasion mechanisms.

## Introduction

*Brassica tournefortii* Gouan (1773) is an annual species from the family Brassicaceae. Native to the Mediterranean region, this species has been introduced to various countries, including the United States, Australia, Colombia, India, and Germany (Bangle et al. 2008; Winkler et al. 2019; Rahmani et al. 2020a, Rahmani et al. 2020b; POWO 2024). Categorized as an invasive plant (Bangle et al. 2008; El-Gawad 2014; Winkler et al. 2019; Rahmani et al. 2020a, Rahmani et al. 2020b), *B. tournefortii* has rapidly expanded into desert areas throughout Australia and the United States, negatively impacting the ecosystems and necessitating proactive management (Chauhan et al. 2006; Bangle et al. 2008; Winkler et al. 2019; Kraus et al. 2020; Rahmani et al. 2020a, Rahmani et al. 2020b. In the Republic of Korea, the species was introduced—likely *via* sand deposition during construction—before 2012 (Kang et al. 2022). Although its invasive potential in South Korea has not been confirmed, the species is spreading into regions with similar environments.

Recent advancements in next-generation sequencing (NGS) technologies have enabled chloroplast genome studies (Nock et al. 2011; Li et al. 2015; Daniell et al. 2016; Li et al. 2016). These tools have significantly advanced biodiversity studies, taxonomy, phylogenetics, and evolutionary biology. Moreover, chloroplast genome data have increasingly been applied to research on invasive species. Genetic diversity analyses using chloroplast-derived simple sequence repeat (SSR) or single-nucleotide polymorphism (SNP) markers have proven instrumental in tracing the origins of invasive species, mapping their dispersal pathways, and identifying genetic factors facilitating their spread (Dowell et al. 2016; Hinsinger and Strijk 2017; Meyer et al. 2017; Winkler et al. 2019). These studies improve our understanding of plant invasion mechanisms and contribute to conservation biology and biodiversity preservation.

Although prior studies have utilized *B. tournefortii* sequences (Winkler et al. 2019; Chhikara et al. 2024), its complete chloroplast genome remains unreported. Hence, this study aimed to sequence the complete chloroplast genome of *B. tournefortii* and analyze its phylogenetic relationships with related species. The findings will provide foundational data for future research across diverse disciplines, including taxonomy, evolutionary biology, and invasive species management.

## Materials and methods

*Brassica tournefortii* was identified and collected by Eun Su Kang from Jarong-ri, Gochang-gun, Jeollabuk-do, South Korea (N 35.43310, E 126.43290). Fresh leaves were dried and preserved in silica gel for DNA extraction. A voucher specimen (KHB1654520) of the sample has been deposited at the National Arboretum Herbarium (KH, http://www.nature.go.kr, Contact: Dong Chan Son, sdclym@korea.kr). DNA extraction was performed using the DNeasy Plant Mini Kit (Qiagen, Hilden, Germany) per the manufacturer’s protocol. High-purity genomic DNA (gDNA) was obtained by filtration through a 2% agarose gel and sequenced on the Illumina MiSeq platform, producing 301 bp paired-end reads, generating 6,646,160 reads.

The chloroplast genome was assembled using the GetOrganelle toolkit v.1.7.7.1 (Jin et al. 2020) through a *de novo* plastome assembly approach. To ensure structural accuracy and completeness, Bandage v.0.8.1 (Wick et al. 2015) was employed for structural visualization, while read coverage depth was verified using the Draw_SequencingDepth.py script from Ni et al. (2023). Genome annotation was performed with GeSeq (Tillich et al. 2017), and gene arrangement was validated against chloroplast genome data for *Brassica* L. species obtained from NCBI (https://www.ncbi.nlm.nih.gov/). The chloroplast genome map, including intron structures of cis- and trans-splicing genes, was visualized using CPGview (Liu et al. 2023).

Phylogenetic relationships between *B. tournefortii* and closely related species were analyzed using a Maximum Likelihood (ML) approach. Chloroplast genomes from 20 species within the Brassicaceae family, including genera *Brassica*, *Raphanus* L., and *Sisymbrium* L., were used, with *Arabidopsis* as the outgroup. Chloroplast genome sequences were retrieved from the NCBI database; 78 coding sequences (CDSs) were extracted and aligned using MAFFT, integrated in PhyloSuite v.1.2.2 (Zhang et al. 2020). ModelFinder v.2.2.0 (Kalyaanamoorthy et al. 2017) was used to determine the best-fit model for ML analysis with IQ-TREE v.2.1.3 (Nguyen et al. 2015) using the GTR+F + R2 model and 1,000 bootstrap replicates. The phylogenetic tree was visualized using FigTree v.1.4.4 (http://tree.bio.ed.ac.uk/software/figtree). Finally, genomes of *B. tournefortii* and 15 Brassicaceae species were analyzed using IRplus (Díez Menéndez et al. 2023) to compare boundary regions between genomic sections. mVISTA (Frazer et al. 2004) identified variation hotspots using the *B. oleracea* var. *botrytis* L. genome as a reference.

## Results

**Figure 1.**
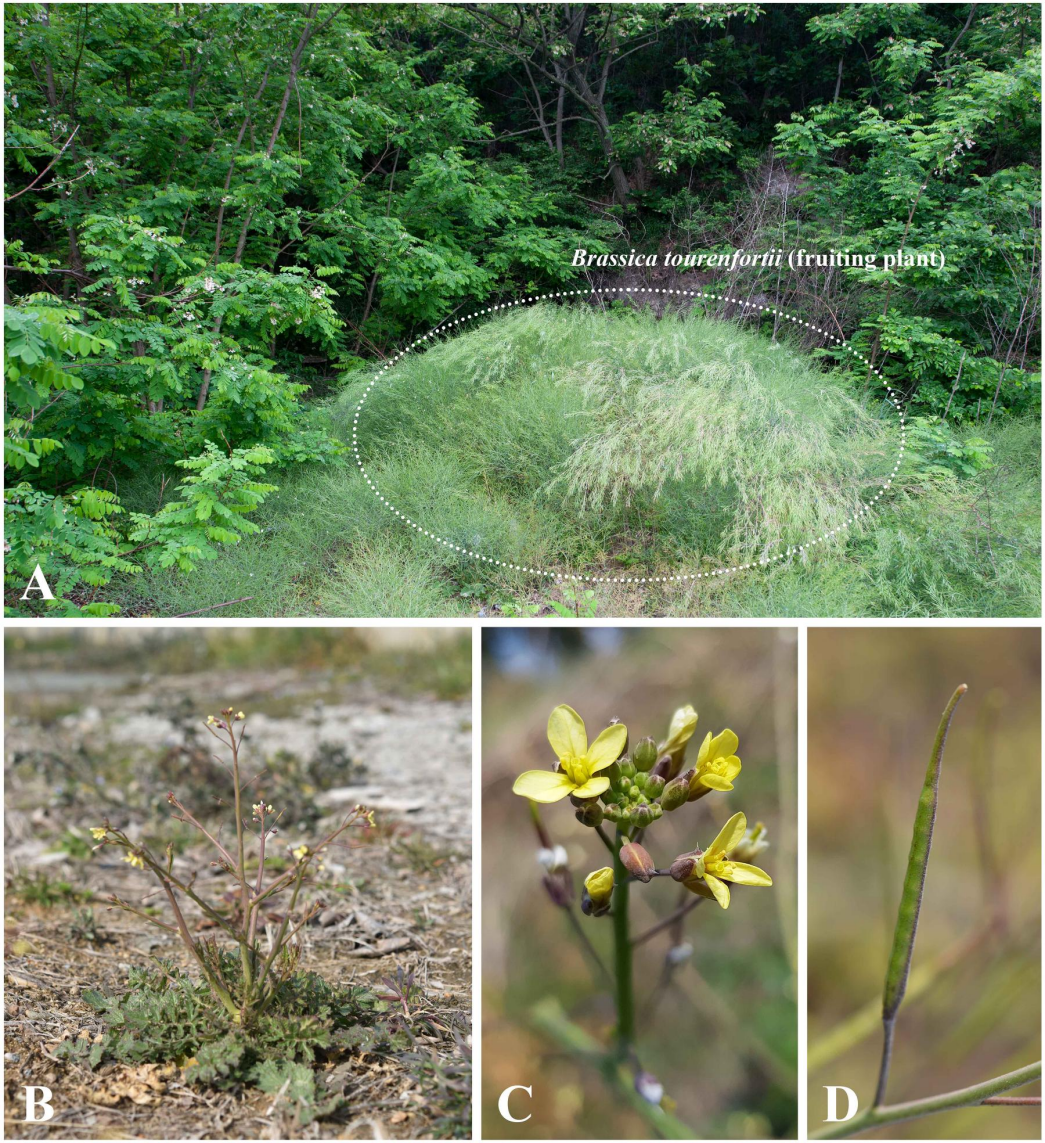
Photographs of *Brassica tournefortii*. The photographs were taken by Eun Su Kang in Gochang-gun, Jeollabuk-do, South Korea. (A) *Brassica tournefortii* is found along mountain edges, coastal areas, roadsides, and disturbed sites, primarily in sandy soil. (B) The plant grows to a height of 1 m, with stems branching basally and distally, and hirsute proximally. (C) Flowers bloom from early spring (February to April), bearing loosely arranged yellow flowers. (D) The fruiting period lasts until May. The fruit has two valves, each with a midvein.

**Figure 2.**
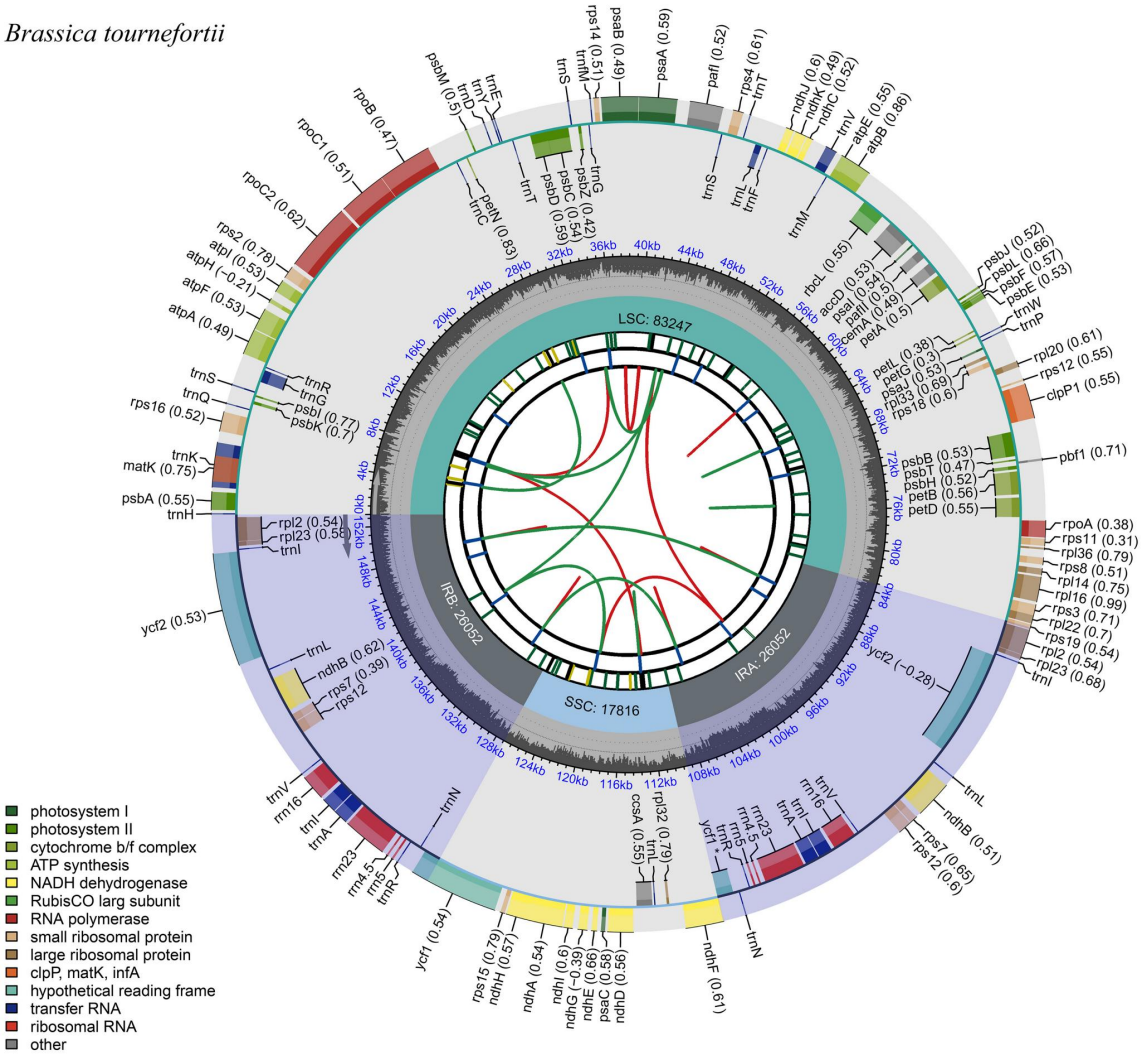
Circular map of *Brassica tournefortii* illustrating the complete chloroplast genome. The map comprises six distinct tracks arranged from the center outward. The innermost track shows dispersed repeats, connected by red and green arcs indicating forward and reverse directions, respectively. The next track highlights long tandem repeats as blue bands, followed by a track marking short tandem repeats (microsatellites), represented as green bands. Another track depicts the GC content across the plastome. The outermost track displays genes as colored boxes, with inner boxes indicating clockwise transcription and outer boxes indicating counterclockwise transcription. Numbers in parentheses following gene names represent optional codon usage bias.

Photographs of *B. tournefortii*, the species used for chloroplast genome analysis, are shown in Figure 1. The chloroplast genome of *B. tournefortii* has a total length of 153,167 bp and a 36.3% GC content (Figure 2), with an average read coverage depth of 1201.76 X (Figure S1). The genome information has been submitted to the NCBI database under accession number PQ783626.

The chloroplast genome has a quadripartite structure common in most angiosperms, including an 83,247 bp large single-copy (LSC) region, a 17,816 bp small single-copy (SSC) region, and two 26,052 bp inverted repeats (IRs).

A total of 129 genes were annotated, including 84 CDSs, 37 tRNAs, and 8 rRNAs. Among these, 17 genes were within the IR regions, comprising 6 CDSs (*ndhB*, *rpl2*, *rpl23*, *rps7*, *rps12, ycf2*), 7 tRNAs (*trnA-UGC*, *trnI-CAU*, *trnI-GAU*, *trnL-CAA*, *trnN-GUU*, *trnR-ACG, trnV-GAC*), and 4 rRNAs (*rrn4.5*, *rrn5*, *rrn16, rrn23*). Eleven cis-splicing genes were identified, including nine genes (*atpF*, *ndhA*, *ndhB*, *petB*, *petD*, *rpl2*, *rpl16*, *rpoC1, rps16*) with a single intron, and two (*clpP1, pafI*) with two introns (Figure S2). Additionally, the trans-splicing gene *rps12* was identified with a single intron spanning the LSC and IR regions, with the 3′ end in the LSC region and the 5′ end in the IR region (Figure S3).

**Figure 3.**
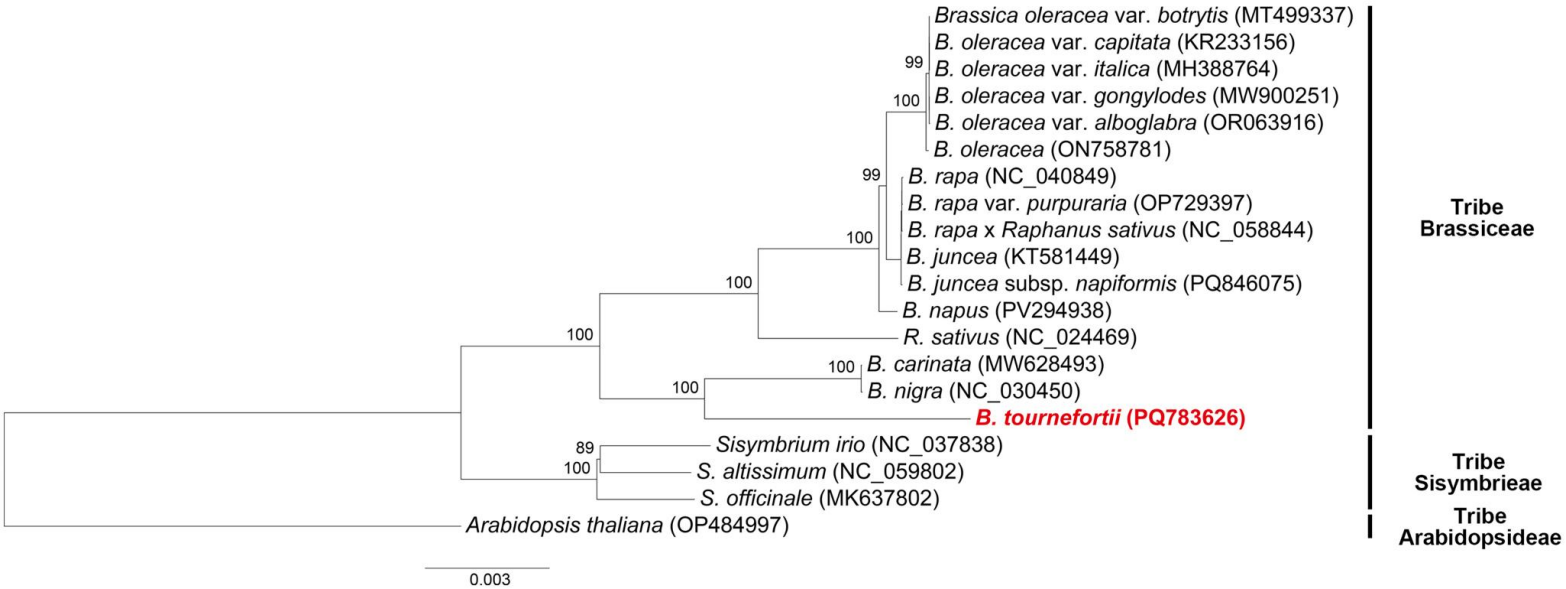
Maximum likelihood phylogenetic tree of 20 species within Brassicaceae. The tree was constructed based on the concatenated 78 CDSs of chloroplast genomes from 16 species from the Brassiceae tribe, 3 from the Sisymbrieae tribe, and 1 outgroup species from the Arabidopsideae tribe. Numbers on the nodes indicate bootstrap proportions: *Brassica oleracea* var. *botrytis* MT499337 (unpublished), *B. oleracea* var. *capitata* KR233156 (Seol et al. 2017), *B. oleracea* var. *italica* MH388764 (Zia et al. 2022), *B. oleracea* var. *gongylodes* MW900251 (Zhao et al. 2024), *B. oleracea* var. *alboglabra* OR063916 (Wang et al. 2023), *B. oleracea* ON758781 (Chen et al. 2021), *B. rapa* NC_040849 (unpublished), *B. rapa* var. *purpuraria* NC_058844 (Gong et al. 2025), *B. rapa* x *Raphanus sativus* NC_058844 (unpublished), *B. juncea* KT581449 (Prabhudas et al. 2016), *B. juncea* subsp. *napiformis* PQ846075 (unpublished), *B. napus* PV294938 (unpublished), *R. sativus* NC_024469 (Jeong et al. 2014), *B. carinata* MW628493 (Zhu et al. 2021), *B. nigra* NC_030450 (Seol et al. 2017), *B. tournefortii* PQ783626 (present study), *Sisymbrium irio* NC_037838 (Kawanabe et al. 2018), *S. altissimum* NC_059802 (Xu and Lin 2021), *S. officinale* MK637802 (unpublished), *Arabidopsis thaliana* OP484997 (unpublished).

ML phylogenetic analysis revealed that *Sisymbrium* species (tribe Sisymbrieae) formed a monophyletic clade, diverging first from *Brassica* and *Raphanus* species (tribe Brassiceae) (Figure 3). Within *Brassica*, species were divided into two groups. *Brassica tournefortii* clustered with *B. carinata* A.Braun and *B. nigra* (L.) W.D.J.Koch, diverging earlier than *Raphanus sativus* L. and other *Brassica* species. Subsequently, *R. sativus* branched, while remaining *Brassica* species formed the final group.

Comparative genomic analysis revealed no significant differences in the IR and LSC or SSC boundary regions across most species (Figure S4). Similarly, mVISTA analysis indicated high similarity among species, with coding regions exhibiting greater conservation than noncoding regions (Figure S5).

## Discussion

This study determined the chloroplast genome sequence of *B. tournefortii* and analyzed its structural features and gene content. The chloroplast genome maintains structural integrity and gene order consistency comparable to other closely related Brassicaceae species.

The ML analysis results aligned with previously reported ML and Neighbor-Joining analyses findings (Yang et al. 2002; Jeong et al. 2014; Seol et al. 2017; Qiao et al. 2020). *Brassica tournefortii* exhibited a close phylogenetic relationship with *B. carinata* and *B. nigra*. In contrast to the *Sisymbrium* group, which formed a monophyletic clade, *R. sativus* appeared as a paraphyletic group within the *Brassica* genus, likely due to its hybrid origin involving *B. nigra* and *B. rapa* L. or *B. oleracea* (Yang et al. 2002; Jeong et al. 2014; Seol et al. 2017; Qiao et al. 2020).

Meanwhile, although *Brassica* encompasses numerous commercially important species (Li et al. 2017), its evolutionary origins remain unresolved, with phylogenetic relationships among species/subspecies incompletely defined (Li et al. 2017). These aspects can be resolved by analyzing the chloroplast genomes of *Brassica* species alongside structural comparisons and phylogenetic investigations.

In this study, however, is limited in scope, as it includes only 15 *Brassica* species, which may not fully represent the global diversity of the genus. Therefore, further research is required with a broader range of *Brassica* species genomes and more detailed comparative and phylogenetic analyses to better understand their evolutionary relationships.

Nonetheless, this study provides a comprehensive chloroplast genome dataset for *B. tournefortii*. These findings support research on *Brassica* phylogeny, evolution, species identification, and invasion, with applications in food and medicine.

## Supplementary Material

Certificate_of_editing.pdf

Supplementary figures.docx

## Data Availability

The complete chloroplast genome sequence data for *B. tournefortii* is available in GenBank of NCBI (https://www.ncbi.nlm.nih.gov/nuccore/PQ783626) under accession number PQ783626. The associated BioProject, Bio-Sample, and SRA numbers are PRJNA1199875, SAMN45889031, and SRR31757948, respectively.
