# Supplementary material for: Chloroplast genome sequencing and phylogenetic analysis of Brassica tournefortii Gouan (Brassicaceae)
Source: Mitochondrial DNA B Resour. 2025 Jul 11;10(8):703–8. doi: 10.1080/23802359.2025.2529842 (PMC12258233; doi:10.1080/23802359.2025.2529842)
Supplement: Supplementary figures.docx [file TMDN_A_2529842_SM5279.docx]

**Supplemental material**

**Figure S1.** Sequencing depth and coverage map of the chloroplast genome assembly of *Brassica tournefortii*. The horizontal axis represents the base position of the plastome, and the vertical axis indicates the sequencing depth corresponding to each base. This figure was enerated using the Draw_SequencingDepth.py script provided by Ni et al. (2023).

**Figure S2.** Schematic representation of the cis-splicing genes in the chloroplast genome of *Brassica tournefortii*. Exons are shown in black, introns in white, and arrows indicate the gene’s sense direction. This figure was enerated using CPGView (Liu et al. 2023).

**Figure S3.** Schematic representation of the trans-splicing gene *rps12* in the chloroplast genome of *Brassica tournefortii*. The arrow indicates the gene's sense direction. This figure was enerated using CPGView (Liu et al. 2023).

**Figure S4.** Comparison of the boundary regions of LSC, IR, and SSC in the chloroplast genomes of 16 species from Tribe Brassiceae: *Brassica oleracea* var. *botrytis* MT499337 (unpublished), *B. oleracea* var. *capitata* KR233156 (Seol et al. 2017), *B. oleracea* var. *italica* MH388764 (Zia et al. 2022), *B. oleracea* var. *gongylodes* MW900251 (Zhao et al. 2024), *B. oleracea* var. *alboglabra* OR063916 (Wang et al. 2023), *B. oleracea* ON758781 (Chen et al. 2021), *B. rapa* NC_040849 (unpublished), *B. rapa* var. *purpuraria* NC_058844 (Gong et al. 2025), *B. rapa* x *Raphanus sativus* NC_058844 (unpublished), *B. juncea* KT581449 (unpublished), *B. juncea* subsp. *napiformis* PQ846075 (unpublished), *B. napus* PV294938 (unpublished), *R. sativus* NC_024469 (Jeong et al. 2014), *B. carinata* MW628493 (Zhu et al. 2021), *B. nigra* NC_030450 (Seol et al. 2017), *B. tournefortii* PQ783626 (present study). The junction between LSC and IRb is abbreviated as JLB, the junction between SSC and IRb as JSB, the junction between SSC and IRa as JSA, and the junction between LSC and IRa as JLA.

**Figure S5.** Aligned sequence plots for the chloroplast genomes using the *Brassica oleracea* var. *botrytis* chloroplast genome as a reference for 16 species from Tribe Brassiceae: *Brassica oleracea* var. *botrytis* MT499337 (unpublished), *B. oleracea* var. *capitata* KR233156 (Seol et al. 2017), *B. oleracea* var. *italica* MH388764 (Zia et al. 2022), *B. oleracea* var. *gongylodes* MW900251 (Zhao et al. 2024), *B. oleracea* var. *alboglabra* OR063916 (Wang et al. 2023), *B. oleracea* ON758781 (Chen et al. 2021), *B. rapa* NC_040849 (unpublished), *B. rapa* var. *purpuraria* NC_058844 (Gong et al. 2025), *B. rapa* x *Raphanus sativus* NC_058844 (unpublished), *B. juncea* KT581449 (unpublished), *B. juncea* subsp. *napiformis* PQ846075 (unpublished), *B. napus* PV294938 (unpublished), *R. sativus* NC_024469 (Jeong et al. 2014), *B. carinata* MW628493 (Zhu et al. 2021), *B. nigra* NC_030450 (Seol et al. 2017), *B. tournefortii* PQ783626 (present study).
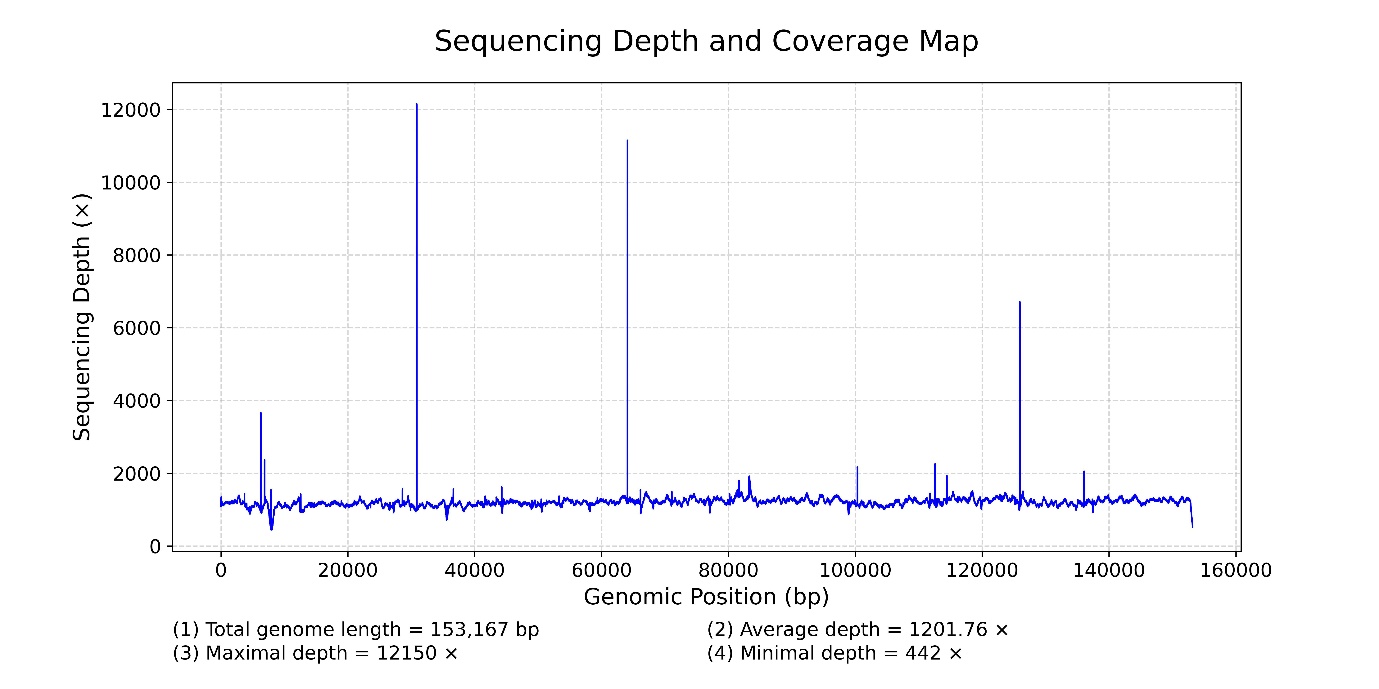


**Figure S1.** Sequencing depth and coverage map of the chloroplast genome assembly of *Brassica tournefortii*. The horizontal axis represents the base position of the plastome, and the vertical axis indicates the sequencing depth corresponding to each base. This figure was generated using the Draw_SequencingDepth.py script provided by Ni et al. (2023).


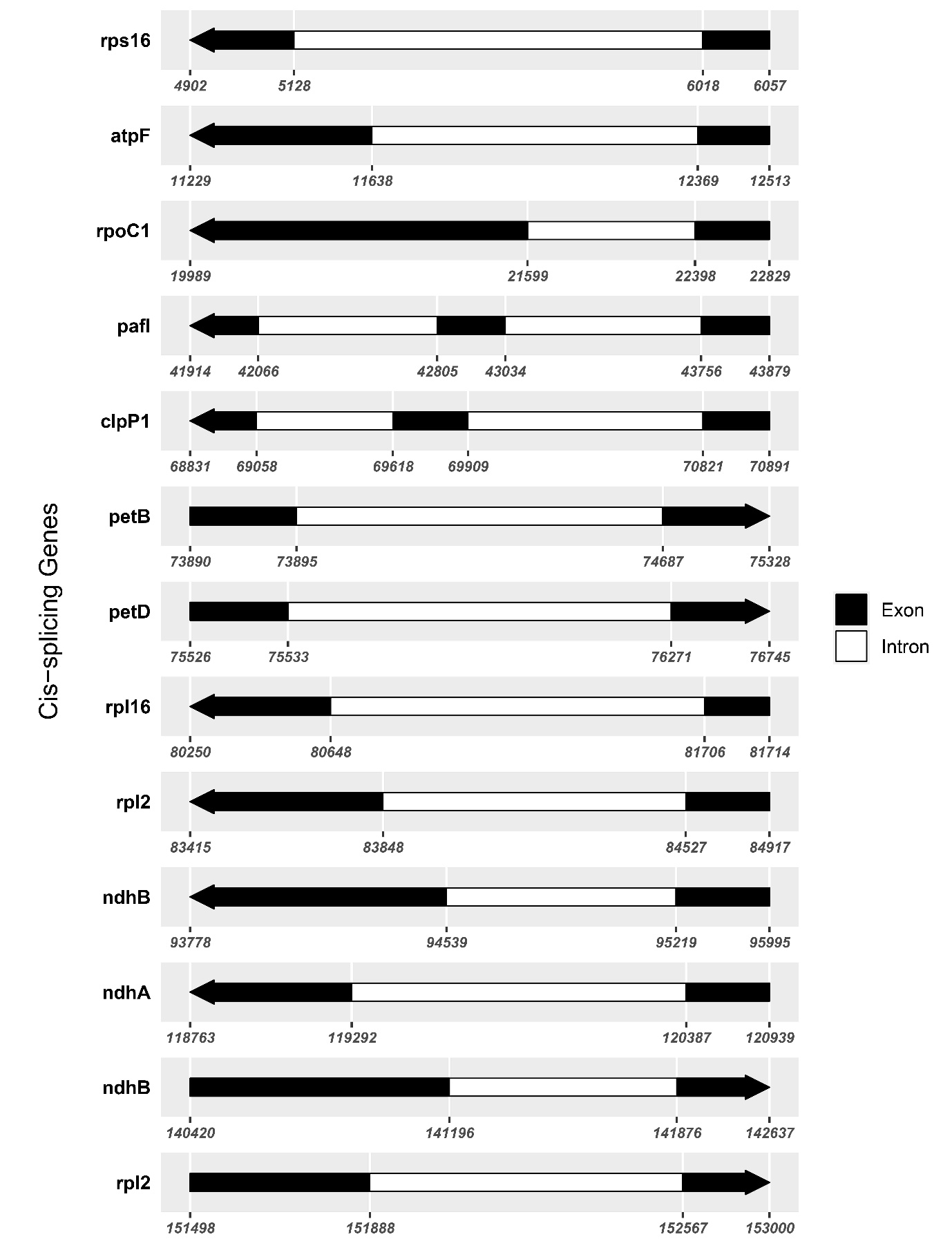


**Figure S2.** Schematic representation of the cis-splicing genes in the chloroplast genome of *Brassica tournefortii*. Exons are shown in black, introns in white, and arrows indicate the gene’s sense direction. This figure was enerated using CPGView (Liu et al. 2023).


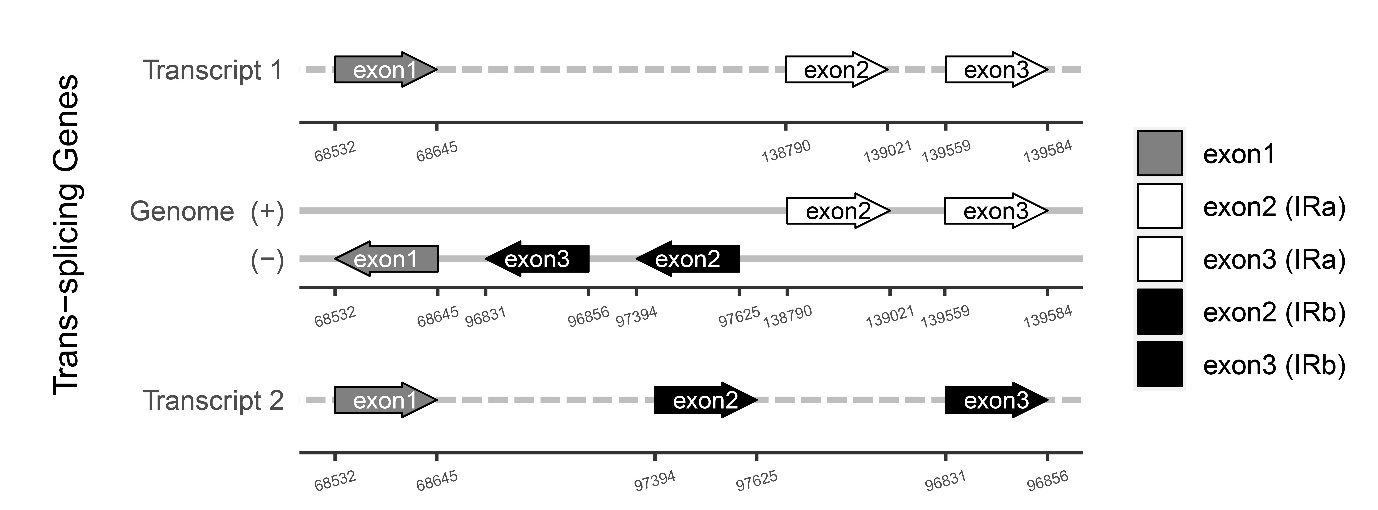
**Figure S3.** Schematic representation of the trans-splicing gene *rps12* in the chloroplast genome of *Brassica tournefortii*. The arrow indicates the gene's sense direction. This figure was enerated using CPGView (Liu et al. 2023).


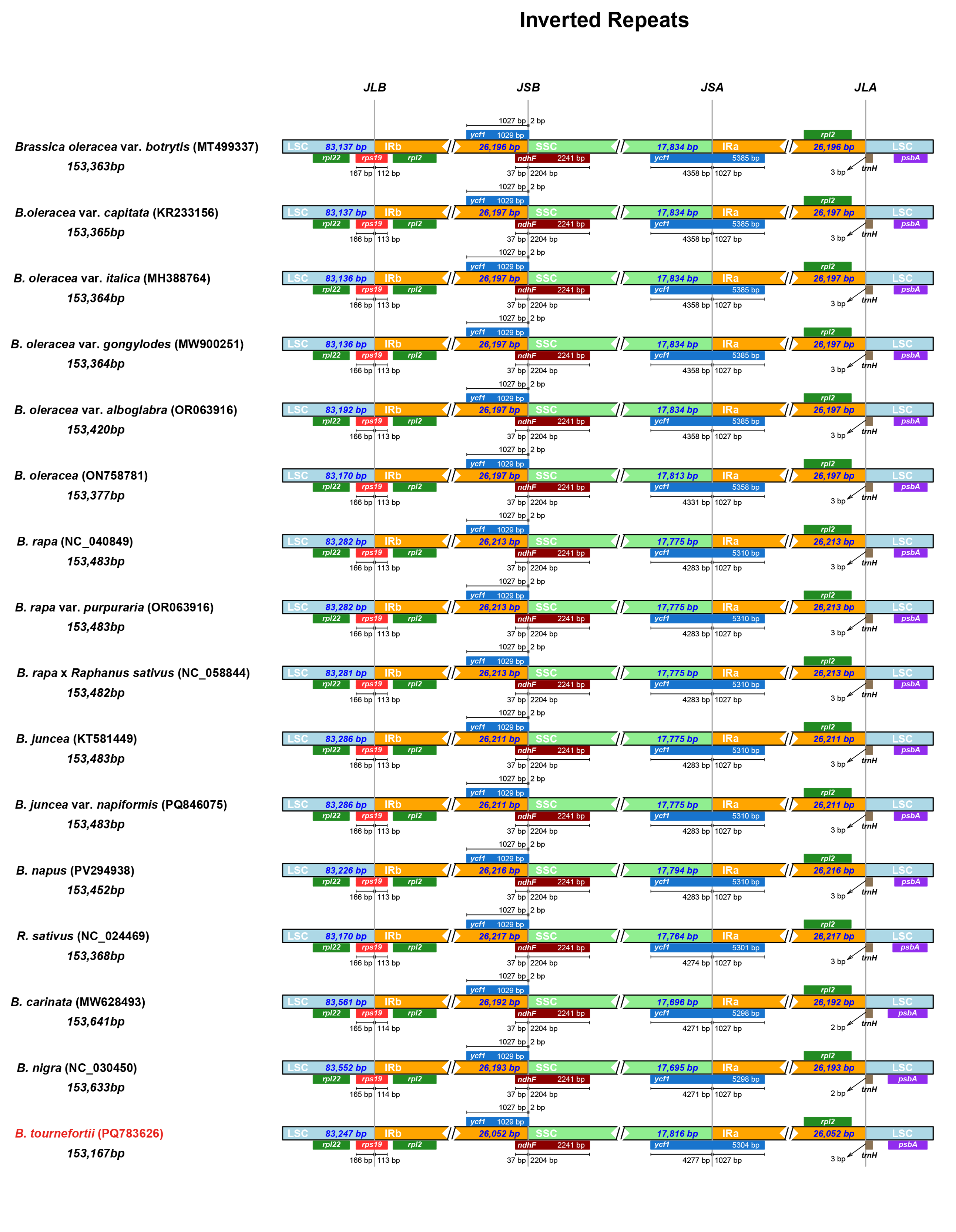


**Figure S4.** Comparison of the boundary regions of LSC, IR, and SSC in the chloroplast genomes of 16 species from Tribe Brassiceae: *Brassica oleracea* var. *botrytis* MT499337 (unpublished), *B. oleracea* var. *capitata* KR233156 (Seol et al. 2017), *B. oleracea* var. *italica* MH388764 (Zia et al. 2022), *B. oleracea* var. *gongylodes* MW900251 (Zhao et al. 2024), *B. oleracea* var. *alboglabra* OR063916 (Wang et al. 2023), *B. oleracea* ON758781 (Chen et al. 2021), *B. rapa* NC_040849 (unpublished), *B. rapa* var. *purpuraria* NC_058844 (Gong et al. 2025), *B. rapa* x *Raphanus sativus* NC_058844 (unpublished), *B. juncea* KT581449 (unpublished), *B. juncea* subsp. *napiformis* PQ846075 (unpublished), *B. napus* PV294938 (unpublished), *R. sativus* NC_024469 (Jeong et al. 2014), *B. carinata* MW628493 (Zhu et al. 2021), *B. nigra* NC_030450 (Seol et al. 2017), *B. tournefortii* PQ783626 (present study). The junction between LSC and IRb is abbreviated as JLB, the junction between SSC and IRb as JSB, the junction between SSC and IRa as JSA, and the junction between LSC and IRa as JLA.

**
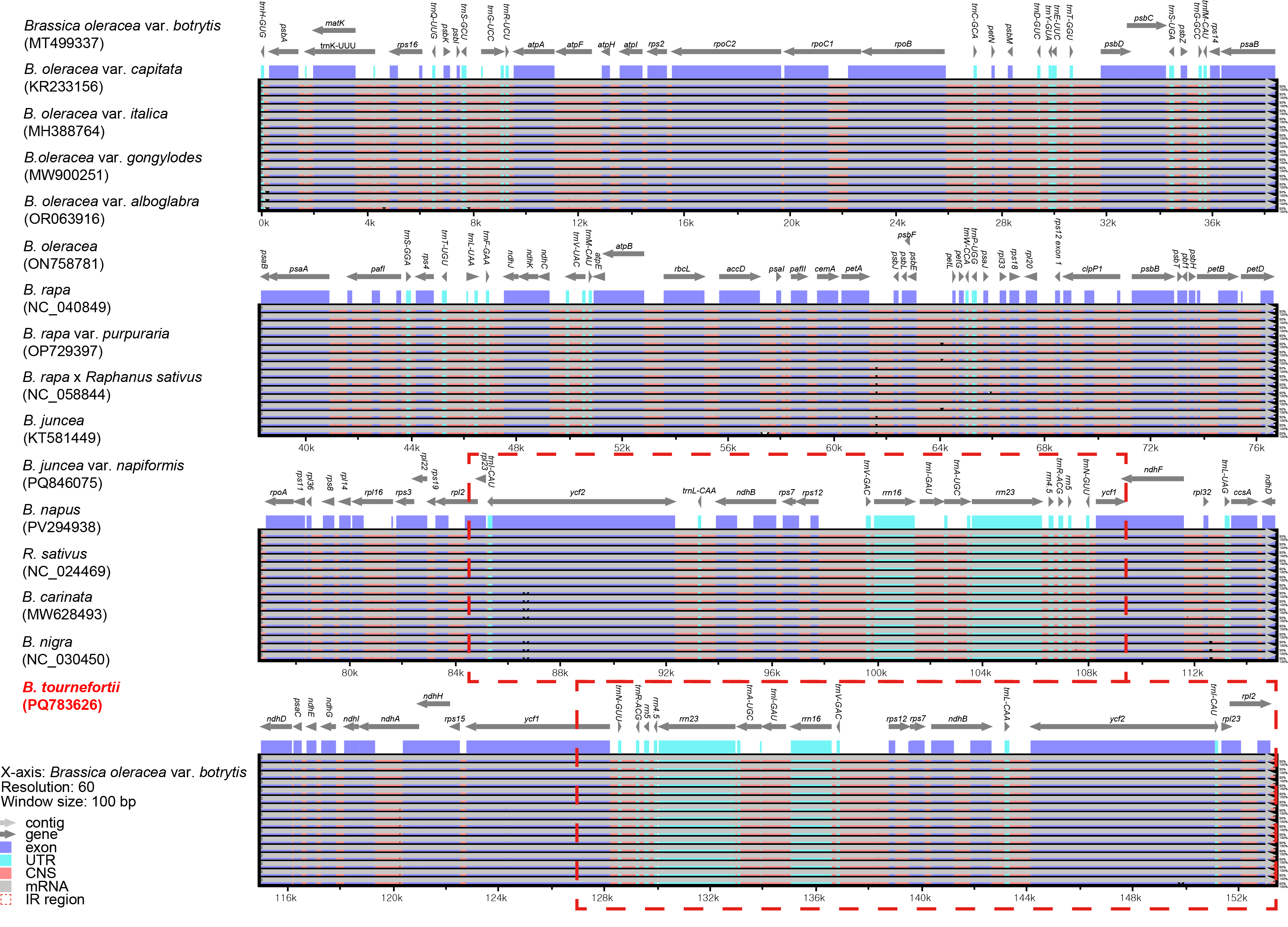
Figure S5.** Aligned sequence plots for the chloroplast genomes using the *Brassica oleracea* var. *botrytis* chloroplast genome as a reference for 16 species from Tribe Brassiceae: *Brassica oleracea* var. *botrytis* MT499337 (unpublished), *B. oleracea* var. *capitata* KR233156 (Seol et al. 2017), *B. oleracea* var. *italica* MH388764 (Zia et al. 2022), *B. oleracea* var. *gongylodes* MW900251 (Zhao et al. 2024), *B. oleracea* var. *alboglabra* OR063916 (Wang et al. 2023), *B. oleracea* ON758781 (Chen et al. 2021), *B. rapa* NC_040849 (unpublished), *B. rapa* var. *purpuraria* NC_058844 (Gong et al. 2025), *B. rapa* x *Raphanus sativus* NC_058844 (unpublished), *B. juncea* KT581449 (unpublished), *B. juncea* subsp. *napiformis* PQ846075 (unpublished), *B. napus* PV294938 (unpublished), *R. sativus* NC_024469 (Jeong et al. 2014), *B. carinata* MW628493 (Zhu et al. 2021), *B. nigra* NC_030450 (Seol et al. 2017), *B. tournefortii* PQ783626 (present study).
